# Fast Diffusion-Based Metabolomics Profiling by 3D Pure Shift HSQC-DOSY NMR with COLMARvista

**DOI:** 10.1021/acs.analchem.6c01028

**Published:** 2026-08-11

**Authors:** R. Cabrera Allpas, K. Lee, D.-W. Li, M. Choo, L. Bruschweiler-Li, R. Brüschweiler

**Affiliations:** † Department of Chemistry and Biochemistry, 2647 The Ohio State University, Columbus, Ohio 43210, United States; ‡ Department of Chemistry, University of North Carolina at Chapel Hill, Chapel Hill, North Carolina 27599, United States; § Department of Biological Chemistry and Pharmacology, The Ohio State University, Columbus, Ohio 43210, United States

## Abstract

Peak overlap is a major obstacle for comprehensive metabolite profiling in NMR-based metabolomics. It is most severe for 1D ^1^H NMR spectra but can also persist for 2D NMR. Here, we demonstrate how metabolite-specific translational diffusion coefficients obtained from a pure shift pseudo-3D HSQC-DOSY experiment can resolve metabolite ambiguities caused by (near-)­degenerate chemical shifts. In addition to the NMR pulse sequence, a novel pseudo-3D processing module implemented in COLMARvista is presented, which is a JavaScript-based standalone software for the automated processing, peak picking, quantification, and visualization of pseudo-3D HSQC-DOSY data sets. As is demonstrated for mouse urine, *Pseudomonas aeruginosa* biofilm, and a carbohydrate mixture, the pseudo-3D real-time pure shift HSQC-DOSY experiment is an effective tool for analyzing complex NMR metabolomics samples. Moreover, it is shown how the increased sensitivity and resolution of this experiment, compared to standard pseudo-3D-DOSY, yield more precise diffusion coefficients of the metabolomic mixture components even when using a minimal set of 2D planes.

## Graphical abstract

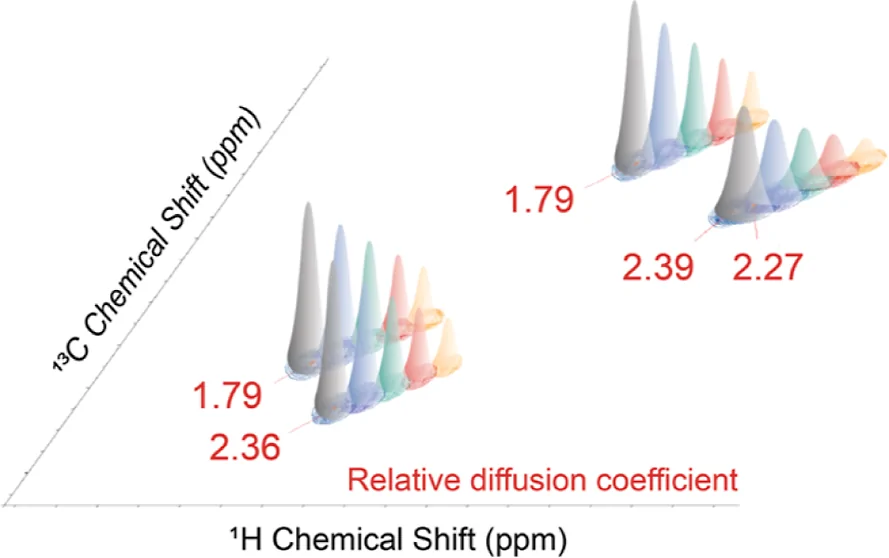

## Introduction

1D ^1^H NMR experiments are commonly used for NMR-based metabolomics studies due to their short measurement times and quantitative accuracy.^1–3^ Both commercial and academic software tools and web servers are available to assist in this process, including Chenomx and Mnova software, BATMAN,^4^ Bayesil,^5^ MagMet,^6^ MetaboLab,^7^ ASICS,^8^ and COLMAR1d.^9^ However, for metabolites with resonances in congested spectral regions, metabolomic profiling by 1D ^1^H NMR spectra can be very challenging due to ambiguous peak identification and assignment to the underlying metabolites. By contrast, 2D NMR experiments, such as 2D ^1^H–^13^C HSQC, 2D ^1^H–^13^C HSQC-TOCSY, and 2D ^1^H–^1^H TOCSY, generally allow a fully untargeted metabolomics approach for the identification and quantification of a maximal number of metabolites in a mixture. These 2D NMR experiments require several hours to acquire, up to a day, but result in much better resolution, which is particularly beneficial for metabolomics studies of highly complex samples,^10–12^ allowing the reliable assignment of most metabolites in a mixture.^13^ Moreover, web servers such as COLMARm^13^ and COLMARq^14^ perform essentially automated assignment using a fully integrated 2D NMR processing, peak picking, and database querying workflow. These web servers query extracted experimental chemical shifts against NMR metabolomics databases such as HMDB,^15^ BMRB,^16^ and COLMAR.^17^ Other workflows, such as COLMAR1d2d,^18^ are available using a metabolite list obtained from COLMARm or COLMARq together with sample-specific chemical shift corrections for the subsequent targeted metabolite identification and quantification from 1D ^1^H NMR experiments by COLMAR1d.^9^ Together, these web servers and software tools enhance, accelerate, and automate metabolite identification and quantification in NMR-based metabolomics.

Unfortunately, even with the help of 2D NMR spectra, the experimental chemical shift information may still be insufficient to unambiguously identify certain metabolites that are affected by cross-peak overlap. Moreover, small chemical shift variations between samples (e.g., due to differences in buffer conditions, such as pH) can further complicate the assignment and analysis of certain metabolites. To overcome such challenges, information that is complementary to ^1^H and ^13^C chemical shifts is needed. For this purpose, translational diffusion coefficients of each metabolite in the solvent buffer are a powerful source of information. They can be obtained from DOSY-type NMR experiments (Diffusion Ordered SpectroscopY) that take advantage of pulsed magnetic field gradients (PFG).^19,20^ DOSY NMR experiments allow the determination of molecular translational diffusion coefficients of metabolites by means of pseudo-2D and pseudo-3D experiments, whereby the PFG strength is systematically varied along the pseudodimension. By fitting the (cross-)­peak signal attenuation caused by translational diffusion to the Stejskal-Tanner (ST) equation,^21^ which describes the signal decay as a function of the experimental gradient strength, gradient duration, and diffusion time, the translational diffusion coefficient *D* can be extracted for each (cross-)­peak.^22^ The resulting diffusion coefficients *D* can then be used to estimate the molecular weight of the compound assigned to a given peak with various approaches having been reported^23^ that relate diffusion coefficients to the molecular weight, including power laws,^24,25^ internal calibration,^26,27^ external calibration,^28,29^ and the SEGWE (Stokes–Einstein–Gierer–Wirtz Estimation) model.^30,31^ The most commonly used DOSY experiment is the pseudo-2D DOSY (i.e., a series of 1D ^1^H spectra whereby the PFG strength is increased between experiments); however, it is often unsuitable for complex metabolomics mixtures because of the above-mentioned severe spectral overlaps. Bi- or multiexponential fitting approaches or inverse Laplace transformation to solve the overlap issue by determining multiple diffusion coefficients from the decay of a single peak^32^ are often underdetermined and unstable. These approaches typically require very high signal-to-noise ratios (S/N) for all contributing mixture components and significantly different diffusion coefficients among the compounds contributing to the overlapped peak. Pseudo-3D DOSY experiments, such as the pseudo-3D HSQC-DOSY, offer higher resolution power^33^ but are substantially more time-consuming due to the need for acquiring a series of entire 2D spectra. For most metabolomic samples at natural ^13^C abundance, achieving adequate sensitivity and resolution requires a large number of scans and *t*_1_-increments, resulting in acquisition times of many hours for a single 2D spectrum, such as a ^1^H–^13^C HSQC experiment.

To overcome these challenges, we introduce a new NMR pulse sequence, which is a real-time pure shift pseudo-3D oneshot ^1^H–^13^C HSQC-DOSY experiment, as an alternative to the standard 3D oneshot HSQC-DOSY experiment.^33^ The real-time pure shift HSQC improves both sensitivity and resolution by eliminating homonuclear vicinal ^3^ *J*(^1^H–^1^H)-splittings along the ^1^H detection dimension, thereby considerably simplifying the resulting spectrum. Moreover, we apply nonuniform sampling (NUS) along the indirect dimension to further improve the resolution while keeping the total acquisition time reasonably short.

For the analysis of the resulting pseudo-3D spectrum, we implemented a new module in the latest version of COLMARvista software (https://github.com/lidawei1975/colmarvista),^34^ which enables convenient pseudo-3D fitting of cross-peak intensities obtained by DEEP Picker^35^ to extract a unique diffusion coefficient for each cross-peak. COLMARvista also allows visualization of the fitting quality and the distribution of diffusion coefficients across the entire spectrum. We demonstrate the new pulse sequence and analyze the results in COLMARvista and COLMARm for a sucrose-glucose mixture, *Pseudomonas aeruginosa* biofilm, and mouse urine.

## Materials and Experiments

A 2.5% (w/v) carbohydrate sample and a 1 mM sample were prepared using standard compounds following the same preparation protocol. These two concentrations were selected to allow comparative analysis under different sample conditions. Sucrose and glucose (Sigma-Aldrich) were dissolved in 180 μL of 99.9% D_2_O, followed by the addition of 20 μL of 500 mM sodium phosphate buffer (prepared in D_2_O) and 2 μL of DSS (4,4-dimethyl-4-silapentane-1-sulfonic acid in D_2_O) as an internal chemical shift reference with a final pH of 7.2. For the 2.5% sample, the final concentration of DSS in the sample was 2 mM, whereas for the 1 mM sample, the final concentration of DSS was 0.5 mM. The aliquots of the final samples were then transferred to 3 mm NMR tubes for data acquisition. The methodology to prepare the *P. aeruginosa* biofilm sample and the mouse urine sample was done as previously described.^18^

The following multidimensional NMR experiments were performed for all 3 samples on an 850 MHz ^1^H frequency Bruker Ascend magnet equipped with an Avance III HD console and a triple resonance inverse cryoprobe: (i) a conventional pseudo-2D DOSY experiment (with pulse sequence *ledbpgppr2s*), (ii) a pseudo-3D oneshot HSQC-DOSY experiment,^33^ and (iii) the new pure shift pseudo-3D HSQC-DOSY experiment. Before acquiring the experiments, the maximum gradient strength was calibrated with the doped water test sample, obtaining a diffusion coefficient of 1.91 × 10^–9^ m^2^/s at 298.15 K, and the temperature calibration was checked using a 99.8% Methanol-d4 as the standard reference sample. For all experiments, the length of the ^1^H 90° pulse was calibrated prior to data acquisition. For the conventional DOSY sequence, the gradient strengths were linearly changed between 5% and 95% of the maximum gradient strength (65.7 G/cm) for a total of 12 increments. For the pseudo-3D HSQC-DOSY experiments, the gradient strengths were changed in constant increments between 5% and 80% of the maximum gradient strength for a total of 5 increments. Gradient duration (δ) and diffusion time (Δ) were calibrated and adjusted accordingly, whereby the same values were used for the pseudo-3D HSQC-DOSY sequences to allow a direct comparison of the results (Table S1). For both pseudo-3D HSQC-DOSY experiments, a 12.5% NUS Poisson-gap schedule^36^ was generated by selecting 128 from 1024 uniformly spaced increments along the indirect dimension with 64 scans per increment. With these parameters, the experimental time was 40–43 h for the pseudo-3D HSQC-DOSY experiments for the biofilm and mouse urine samples. This NUS schedule worked well for these complex mixture samples and was not further optimized. For comparison, a uniformly sampled (US) pure shift pseudo-3D HSQC-DOSY experiment was acquired for the biofilm sample in a similar amount of measurement time as the NUS experiment using all 1024 uniformly spaced increments along the indirect dimension with 8 scans per increment.

Processing of the pseudo-2D DOSY experiments was done in Topspin 3.7.0. The analysis and plotting of the pseudo-2D DOSY were done using Dynamics Center 2.8.4. Processing of the pseudo-3D HSQC-DOSY experiments was done in NMRPipe.^37^ The SMILE algorithm^38^ was used to process the spectrum along the indirect (^13^C) NUS dimension. After all planes of the pseudo-3D HSQC-DOSY experiments were processed, the analysis of the data was performed in COLMARvista.

## COLMARvista pseudo3D Fitting Tool and DOSY Analysis Tool

After processing the pseudo-3D spectral data sets in NMRPipe (ft2 frequency domain format), the data were imported into COLMARvista for simultaneous visualization and analysis of all HSQC planes. Specifically, COLMARvista applied the DEEP Picker and Voigt Fitter algorithms to deconvolute the 2D NMR spectra into individual peaks and fit their shapes. After peak picking and fitting of one of the planes (the plane belonging to the lowest gradient field strength with the highest S/N), the COLMARvista pseudo-3D fitting tool was used to deconvolute the rest of the planes. Since peak shapes remain unchanged for different gradient strengths, peak amplitudes are used for the extraction of the diffusion coefficients *D* (vide infra). We also applied Monte Carlo-based error estimation due to thermal random noise and generated the reconstructed spectrum for all the fitted planes for visual inspection. COLMARvista then performed DOSY fitting using the gradient values used in the experiment.

In order to compare how the fitting is affected when using only a subset of the gradients (e.g., only the first and last gradient), the user can select the subset of 2D HSQC planes to be used. We also allow the user to adjust the global diffusion constant scaling factor *f* to obtain correct absolute diffusion coefficients. The equation used for fitting is given by^21^

1 $$I\left(g\right)=I\left(0\right)e^{-D\gamma^{2}\delta^{2}\Delta^{\prime }g^{2}}$$

Which describes the attenuated signal *I*(*g*) relative to *I*(0), where *g* is the corresponding gradient strength, γ is the gyromagnetic ratio of the nuclei being studied (^1^H), δ is the gradient duration, Δ′ is the corrected diffusion delay, and *D* is the diffusion coefficient. For the pseudo-3D oneshot HSQCDOSY experiment, the corrected diffusion delay Δ′ is given in ref 33.

The global scaling factor *f* = $\frac{1}{\gamma^{2}\delta^{2}\Delta^{\prime }}10^{10}$ can then be calculated using the corresponding NMR acquisition parameters, returning a diffusion coefficient in units of 10^–10^ m^2^/s. The graphical user interface of COLMARvista displays the fitting curve together with the value of the diffusion coefficient next to each cross-peak, a table with all fitted intensities, and error estimates.

In this work, diffusion coefficients extracted from urine and biofilm samples were normalized relative to the diffusion coefficient of a metabolite that is commonly found in metabolomics samples (in this study, betaine (glycine betaine)) and has a strong isolated methyl cross-peak that can be accurately quantified. This normalization allows us to quantitatively compare relative diffusion properties across different samples, irrespective of changes in the overall viscosity, which can change due to different sample composition and solvent buffer conditions.

## Results

We first tested the approach for a model mixture with relatively high concentrations of sucrose and glucose (2.5% (w/v) each), to compare the 3D oneshot HSQC-DOSY pulse sequence and our new pure shift pseudo-3D HSQC-DOSY pulse sequence (Figure 1). Figure 1B shows a selected region of the spectrum obtained by the pure shift pseudo-3D HSQC-DOSY pulse sequence, which eliminates most ^1^H–^1^H *J*-splittings, while Figure 1C shows the same spectral region obtained by the oneshot HSQC-DOSY sequence. At these high concentrations and high S/N, the data closely follow a monoexponential decay. For both molecules, there is no significant difference between the averages of the fitted diffusion coefficients (sucrose: 1.79 ± 0.01 vs 1.77 ± 0.05 (×10^–10^ m^2^/s); glucose: 2.32 ± 0.08 vs 2.29 ± 0.11 (×10^–10^ m^2^/s)) between the pure shift pseudo-3D HSQC-DOSY and the oneshot pseudo-3D HSQC-DOSY, respectively.

**Figure 1:**
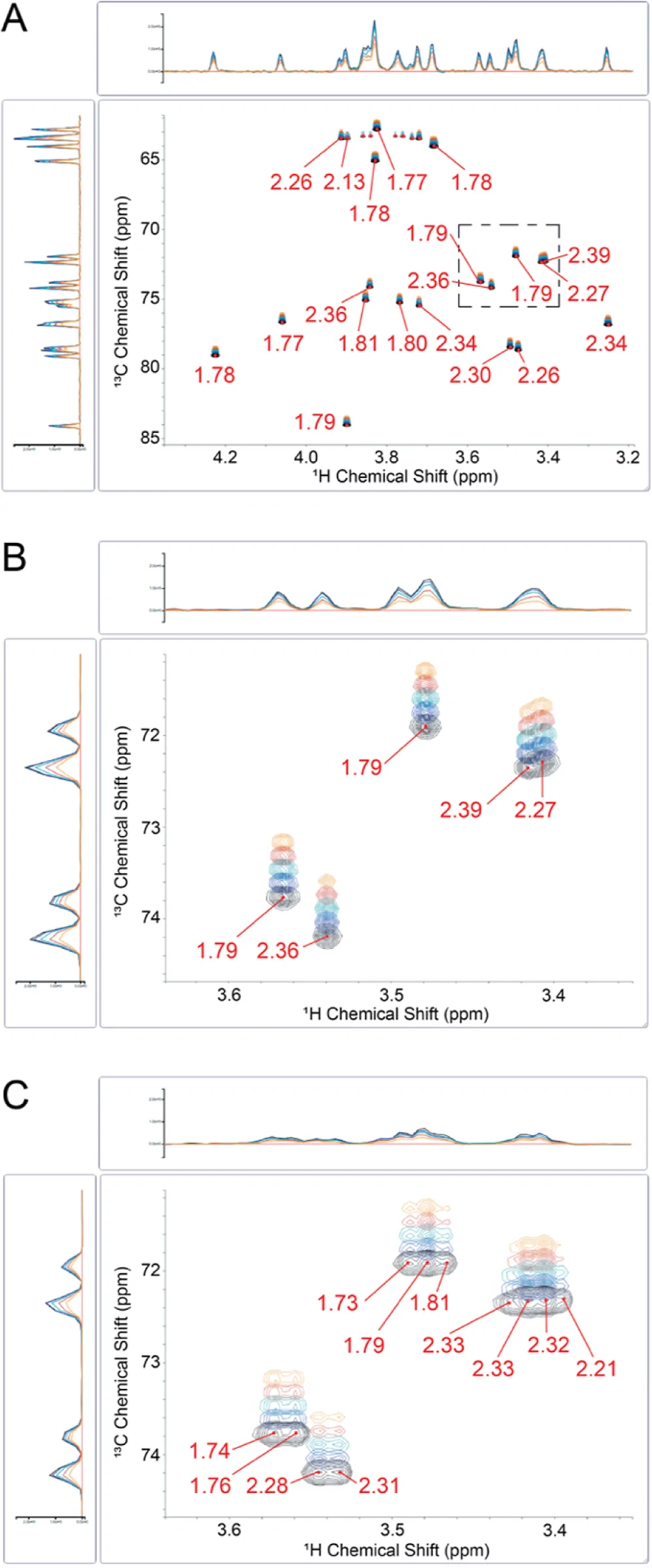
(A) Representative section of the pseudo-3D ^1^H–^13^C HSQC-DOSY spectrum of the sucrose-glucose model mixture (2.5% w/v). The dashed rectangle indicates the region expanded in (B,C). Comparison of the expanded spectral region obtained with (B) the pure shift pseudo-3D HSQC-DOSY sequence and (C) the oneshot pseudo-3D HSQC-DOSY sequence, displayed using COLMARvista, an NMR spectral processing and visualization software. Each cross-peak is labeled in red with its assigned translational diffusion coefficient (*D*) of the corresponding molecule in units of 10^–10^ m^2^/s. Peaks belonging to different gradient strengths are colored differently and, for visualization purposes, they are systematically displaced along the ^13^C chemical shift axis.

The signal-to-noise ratio is substantially higher in the pure shift pseudo-3D HSQC-DOSY experiment since the collapse of cross-peak multiplet structure leads to sensitivity gains of 40%–200%, which can be crucial for metabolites present at low concentration. In the case of the model mixture where sucrose and glucose are at a concentration of 1 mM, the standard deviation is significantly higher for the oneshot sequence: sucrose, 2.01 ± 0.03 × 10^–10^ vs 1.99 ± 0.37 × 10^–10^ m^2^/s and glucose, 2.60 ± 0.21 × 10^–10^ vs 2.66 ± 0.39 × 10^–10^ m^2^/s for the pure shift pseudo-3D HSQC-DOSY sequence vs the oneshot pseudo-3D HSQC-DOSY sequence, respectively. For the pure shift sequence, the standard deviation of glucose increased as well because it does not eliminate geminal ^2^ *J*(^1^H–^1^H)-couplings within methylene groups. As a result, little or no sensitivity gain is observed for the C6 methylene resonances of glucose. After validating the precision of each metabolite’s diffusion coefficient, we assessed the goodness-of-fit. The peak shapes follow in excellent approximation Voigt profiles as evidenced by the agreement between the experimental and fitted peaks illustrated for a congested spectral region of the sucrose-glucose mixture (Figure S2). Figure 2 presents selected DOSY fits for comparison between the pure shift pulse sequence (Figure 2A) and the oneshot (Figure 2B) experiment. The quality of fits for representative peaks of the two DOSY experiments is illustrated in Figure 2C–E. A direct comparison focusing on the H-4′/C-4′ cross-peak of the fructose unit in sucrose is shown in Figure S1, where the collapse of the J-triplet into a singlet in the pure shift experiment leads to a more linear (monoexponential) decay compared to the decays of the individual J-triplet components observed in the oneshot experiment.

**Figure 2:**
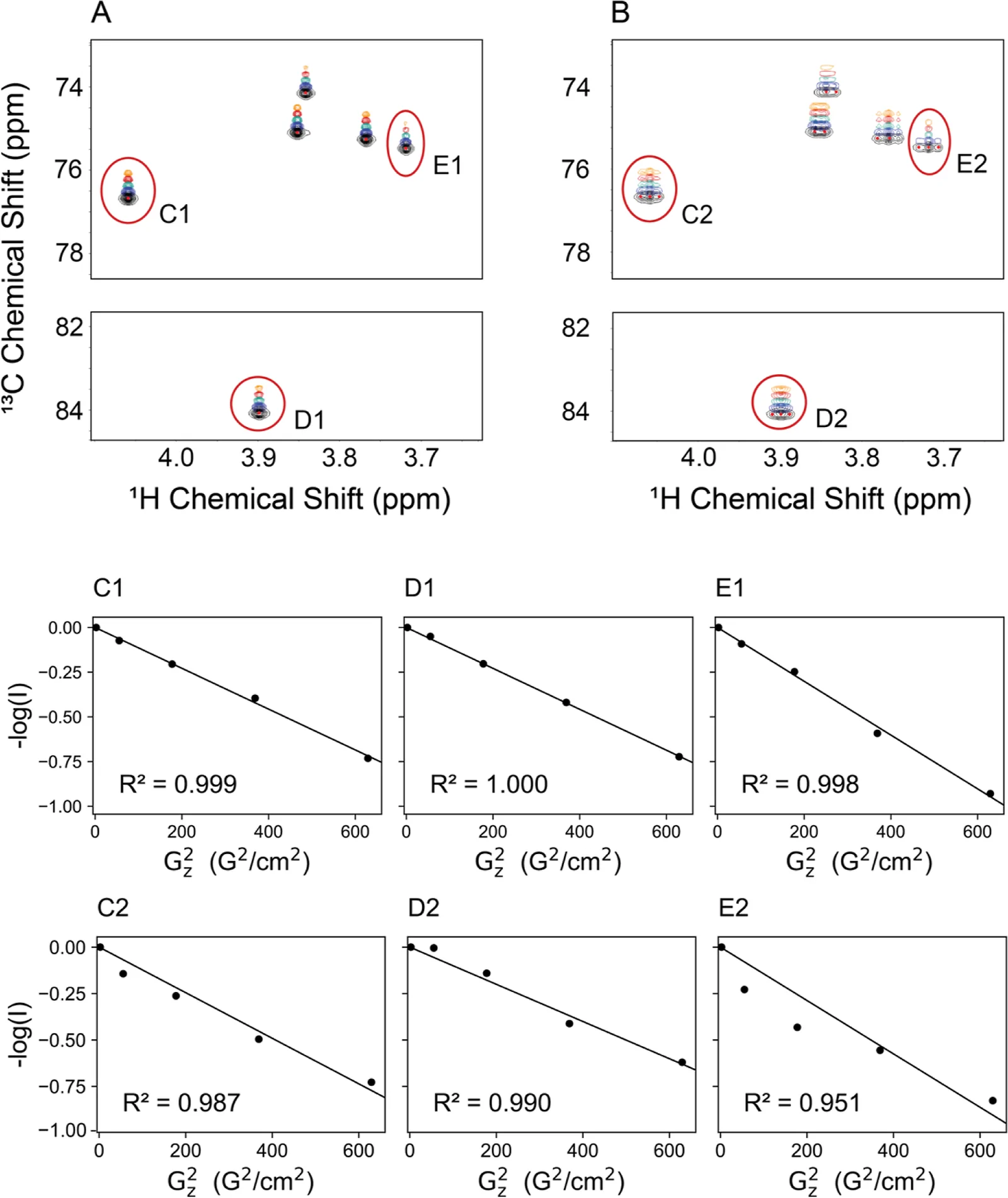
DOSY fitting curves visualized in COLMARvista for the sucrose-glucose model mixture. The top panels display expanded spectral regions obtained using (A) the pure shift pseudo-3D HSQC-DOSY sequence and (B) the oneshot pseudo-3D HSQC-DOSY sequence. Red circles indicate the cross-peaks chosen for diffusion analysis in the plots below, which show the signal decays and their log–linear fits: panels (C1,D1,E1) correspond to the peaks in (A), while panels (C2,D2,E2) correspond to the equivalent peaks in (B) providing a direct comparison of the fit quality between the two experimental methods.

Next, we assessed the pulse sequence performance for two real-world metabolomics samples with high spectral complexity, namely *P. aeruginosa* biofilm and mouse urine. A histogram of the relative diffusion coefficients obtained by the new sequence is presented in Figure 3 after normalizing against the diffusion coefficient of betaine, which was found to be 3.82 × 10^–10^ m^2^/s and 4.05 × 10^–10^ m^2^/s for biofilm and mouse urine, respectively. The diffusion coefficient of betaine was chosen as a reference because of the high intensity of the methyl cross-peak belonging to the three equivalent methyl groups of betaine, as well as for being a common metabolite in both samples. Figure 3A shows that the distribution of relative diffusion coefficients derived from HSQC cross-peaks in the biofilm sample (0.684 ± 0.230), and Figure 3B shows the corresponding distribution for the mouse urine sample (0.739 ± 0.173). The biofilm sample contains a higher fraction of cross-peaks near 0.4 that belong to maltose, maltotetraose, and oxidized glutathione. In contrast, mouse urine shows a higher fraction of cross-peaks near 0.6 belonging to citrate, capryloylglycine, and pantothenate. The resolving power of the diffusion coefficient measurements was assessed based on the intrametabolite coefficient of variation (CV = σ/mean) of diffusion coefficients across multiple cross-peaks of the same metabolite. In the biofilm sample, the CV was 1.22% for trehalose, 1.98% for glucose, and 2.38% for maltose, corresponding to standard deviations of 0.0065, 0.0140, and 0.0100 in relative diffusion coefficients, respectively. Likewise, in the mouse urine sample, the CV was 1.58% for capryloylglycine, 1.61% for hippuric acid, and 3.45% for glucose, corresponding to standard deviations of relative *D* of 0.0093, 0.0119, and 0.0259, respectively. These intrametabolite variations are much smaller than the overall width of the diffusion coefficient distributions of Figure 3 of both biofilm (0.684 ± 0.230) and mouse urine (0.739 ± 0.173), indicating that the dynamic range of relative diffusion coefficients is sufficiently high to help resolve metabolite ambiguities in many cases. Trehalose and maltose in the biofilm sample, despite having the same molecular formula and molecular weight, were well separated by their mean relative diffusion coefficients (0.53 and 0.42, respectively), whereas glucose and hippuric acid in the mouse urine sample remained difficult to distinguish because their mean relative diffusion coefficients were nearly identical (0.75 and 0.74, respectively). This suggests that for the types of high complexity mixtures as those used in this work diffusion information is best used in combination with chemical-shift information. Figure 4 demonstrates correlations between the relative diffusion coefficients obtained in the biofilm and mouse urine samples by the standard pseudo-3D HSQC-DOSY and the pure shift pseudo-3D HSQC-DOSY for the known metabolites detected in these samples.

**Figure 3:**
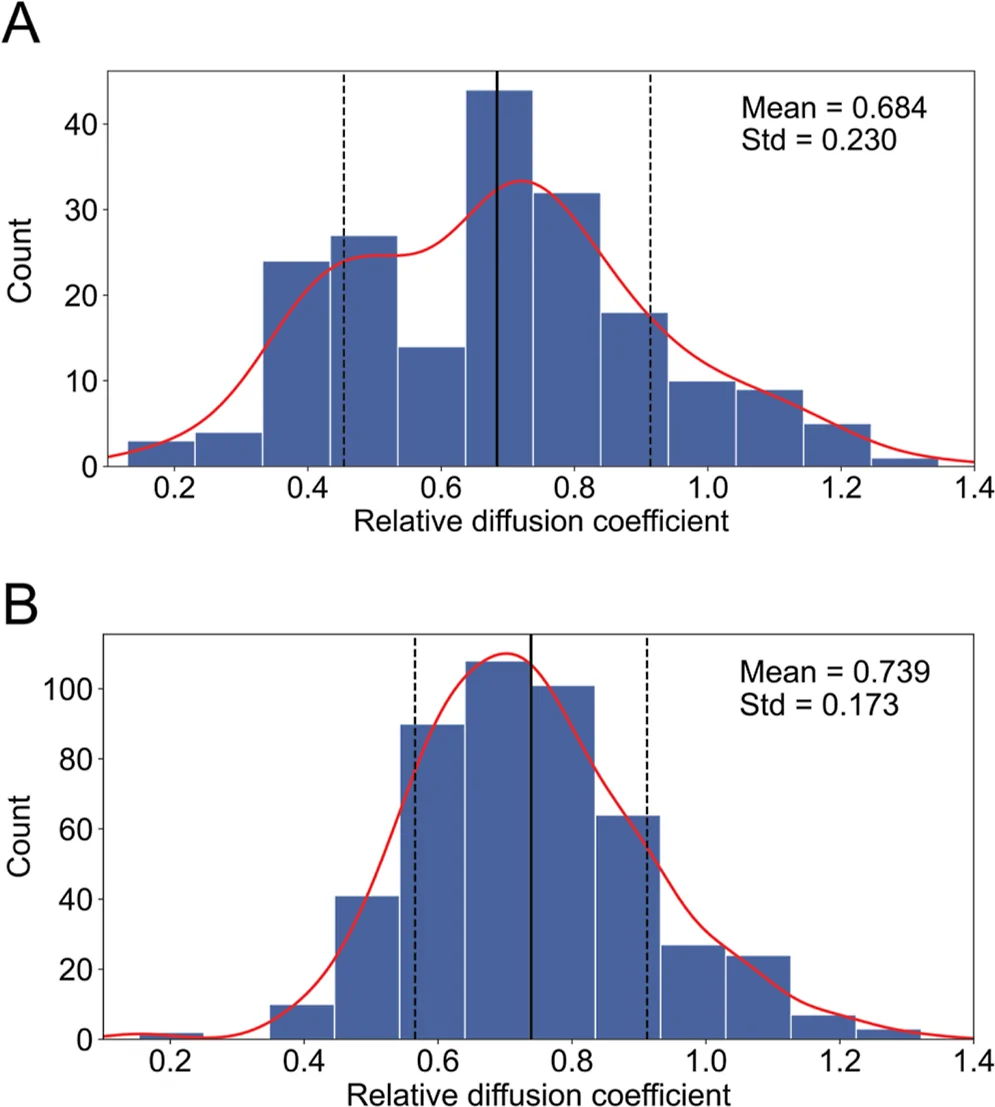
Histograms of relative diffusion coefficients extracted from pure shift pseudo-3D HSQC-DOSY spectra of (A) a biofilm sample and (B) a mouse urine sample. The diffusion coefficient for each HSQC cross-peak was determined and normalized against the reference methyl signal of betaine. The solid vertical line marks the mean value, and the dashed vertical lines indicate the standard deviation (±1 std). The red curves represent the estimated density of the distribution.

**Figure 4:**
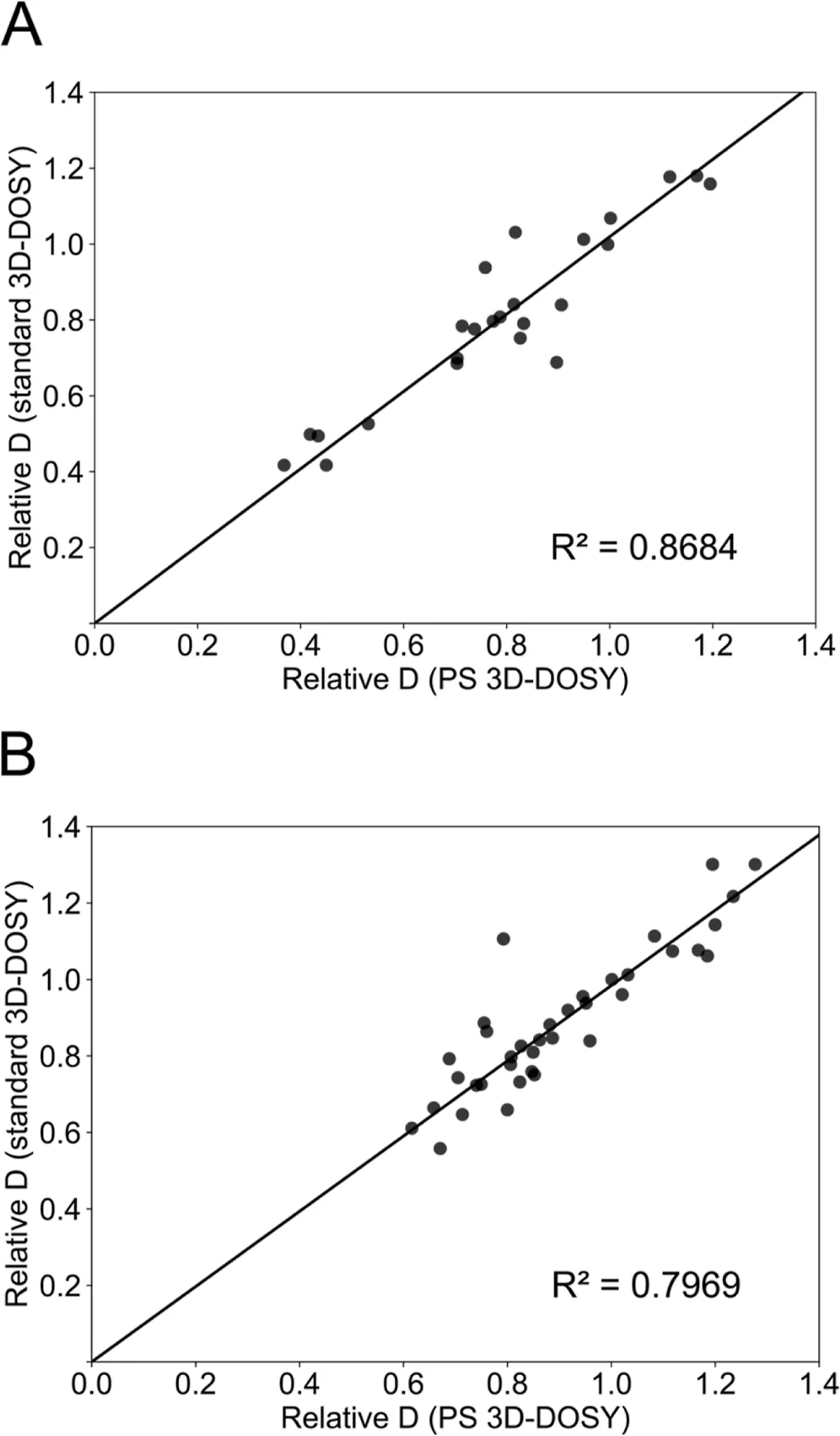
Correlation of relative diffusion coefficients obtained from the oneshot pseudo-3D HSQC-DOSY (*y*-axis) and pure shift pseudo-3D HSQC-DOSY (*x*-axis) experiments. Data are presented for (A) the biofilm sample and (B) the mouse urine sample. Each data point corresponds to a distinct metabolite, where the value corresponds to the average diffusion coefficient of all cross-peaks belonging to that metabolite. The solid line represents the linear regression fit with zero intercept between the two sets of diffusion coefficients with the *R* ^2^ coefficient of determination indicated in each panel.

DOSY-based translational diffusion coefficients directly report on the effective molecular size of molecules.^19,23^ In the context of complex biological mixtures, such information can be very beneficial for the correct identification of certain metabolites whose NMR signals strongly overlap with those of other metabolites of different molecular size, particularly when such information is paired with chemical shift-based identification approaches. Since each plane of the pure shift pseudo-3D HSQC-DOSY experiment corresponds to a 2D HSQC spectrum, the first plane, which has the highest sensitivity, can be directly queried against a metabolomics database for metabolite identification, such as COLMARm. Figure 5 shows how multiple database compounds match within the default chemical shift cutoff the same single cross-peak in the biofilm ^1^H–^13^C HSQC spectrum. According to the database, oxidized glutathione (612.63 g/mol) and gamma-glutamylcysteine (250.27 g/mol) have entirely overlapped cross-peaks and could not be discriminated based on chemical-shift information alone. However, the relative diffusion coefficient of 0.40 determined for the cross peak at (2.155, 28.9) ppm offers important clues. In fact, oxidized glutathione agrees well with this diffusion coefficient, whereas gamma-glutamylcysteine is expected to have a relative diffusion coefficient of 0.60 or higher, which is inconsistent with the DOSY data. To validate the accuracy of the relative diffusion coefficients obtained from the nonuniformly sampled (NUS) experiment, we compared matched peaks between the NUS and uniformly sampled (US) pure-shift pseudo-3D HSQC-DOSY data sets for the biofilm sample. The similar quality of DOSY fits and good agreement suggests that potential artifacts introduced by NUS reconstruction have minimal effect on the derived diffusion coefficients (Figure S3). Representative trehalose-derived cross-peaks used for fitting and relative diffusion coefficient analysis of the NUS and US data sets are shown in Figure S4.

**Figure 5:**
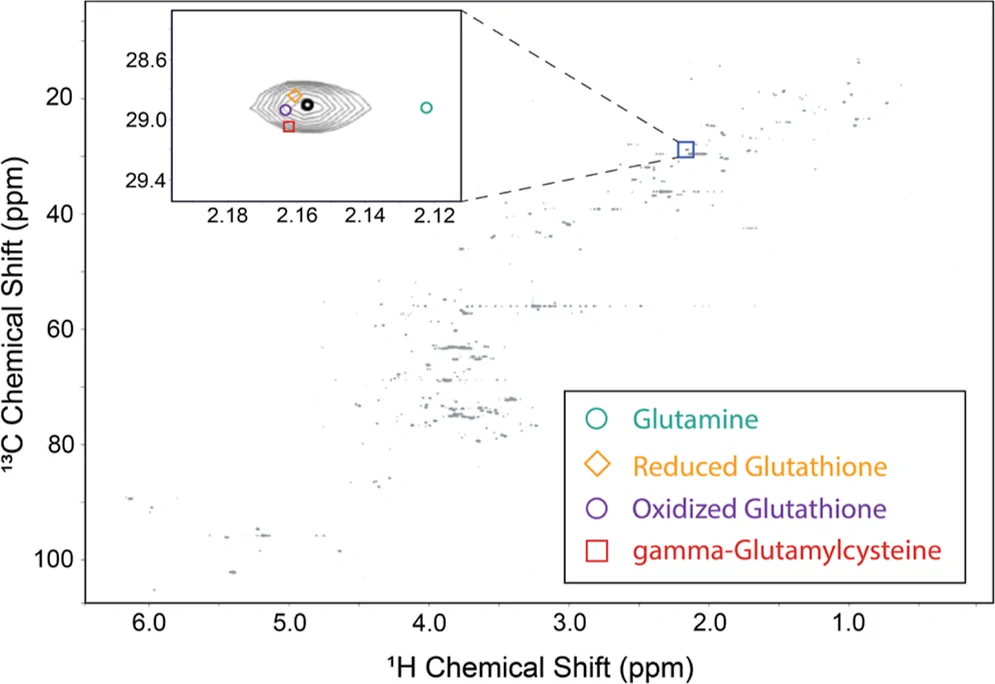
Full spectrum of the first 2D plane extracted from the pure shift pseudo-3D HSQC-DOSY experiment of the biofilm sample. The inset displays a cross-peak successfully isolated by nonuniform sampling (NUS). While COLMARm analysis proposed four candidate metabolites (indicated by symbols) based on chemical shifts alone, the measured relative diffusion coefficient of 0.40 allowed the unique assignment to oxidized glutathione.

Diffusion information can also help discriminate between other candidate compounds with nearly identical chemical shifts. For example, for distinguishing glucose vs glucose-1-phosphate with relative diffusion coefficients of 0.70 vs 0.60 and monosaccharides vs disaccharides, such as glucose vs trehalose, with relative diffusion coefficients of 0.70 vs 0.53. More generally, we anticipate that many metabolites that can occur also in chemically modified form at remote sites, such as phosphorylation (e.g., arginine vs arginine phosphate), acetylation, and possibly even methylation, can be uniquely identified using the pure shift pseudo-3D DOSY approach presented here. Since NMR chemical shifts are local probes of molecular structure, and translational diffusion reflects a global molecular property, the two parameters synergistically complement each other for metabolite identification. The pseudo-3D DOSY serves a similar purpose as ion mobility-derived collision cross sections (CCS) in mass spectrometry.^39^ In practice, the relative diffusion coefficients are most informative when their difference between candidate metabolites is larger than the peak-to-peak variation observed within the same metabolite. Further illustrations of how diffusion data can be obtained with COLMARvista for each cross-peak of pure shift pseudo-3D HSQC-DOSY spectra are provided for biofilm and mouse urine in the Supporting Information (Figures S5 and S6).

## Discussion

Our results demonstrate that the pure shift pseudo-3D HSQC-DOSY yields both superior sensitivity and resolution, which directly translates into improved accuracy of the fitted, cross-peak specific diffusion coefficients. Considering how critical the S/N is in many metabolomics applications, pure shift pseudo-3D HSQC-DOSY allows the more quantitative determination of diffusion coefficients for more metabolites in comparison with standard pseudo-3D HSQC-DOSY experiments. The quantitative comparison between the two types of experiments in Figure 4 demonstrates that the extracted diffusion coefficients can notably differ, which can be attributed to weak cross-peaks in the traditional experiment, leading to larger uncertainties in the corresponding diffusion coefficients (Figure S7). In other cases, entire cross-peaks were missing in the traditional pseudo-3D HSQC-DOSY spectra that were clearly present in the pure shift experiment, thus preventing quantitative comparison.

One drawback of the real-time pure shift HSQC experiment is that it retains the geminal ^2^ *J*(^1^H–^1^H) splittings of diastereotopic CH_2_ groups, which leads to lower sensitivity and larger uncertainties in the resulting diffusion coefficients for these cross-peaks. In addition, small artifacts due to strong coupling effects can be found in the center between the diastereotopic CH_2_ resonances. Although users need to distinguish these artifacts from real cross-peaks, it is usually not an issue for metabolite identification by COLMARm, as these artifacts are unlikely to match chemical shifts in the COLMAR database when paired with additional 2D NMR spectra, such as TOCSY and HSQC-TOCSY. Artifacts appearing as wiggles arising from chunk acquisition in real-time pure shift experiments, especially those coming from particularly strong cross-peaks, also need to be suppressed. We used a modified BIRD pulse scheme to minimize their effects.^40^ In COLMARvista, the user can conveniently remove such artifacts before proceeding with the pseudo-3D fitting toolkit. The different types of artifacts described above are illustrated in Figure S8.

In the case of our complex mixtures, we used a normalization procedure for the diffusion coefficient, since differences in sample viscosity complicate the use of absolute diffusion coefficients, making it hard to compare the diffusion results obtained for different samples. Instead, by normalizing the diffusion coefficients of all compounds with respect to the diffusion coefficient of a common reference compound, such as betaine, we can determine for each cross-peak and each compound a relative diffusion coefficient, which is transferable between samples that belong to the same cohort. Relative diffusion coefficients greatly help mitigate viscosity effects, although differential solvation or interactions with other mixture components in the sample can potentially affect diffusion coefficients between samples.^41^

A drawback of these pseudo-3D DOSY experiments is the total measurement time needed, which makes it impractical to measure these experiments using all 5 or more PFG increments for each sample for routine metabolomics applications. To address this problem, we compared the fitting results when all five HSQC planes were used versus when only two planes were used (first and last gradient strength only) for both biofilm (Figure 6A) and mouse urine (Figure 6B). The 2-plane fits are well-consistent (*R* ^2^ > 0.90) with the 5-plane fits, with the largest differences observed for peaks with relatively low signal-to-noise. Mouse urine is a significantly more complex mixture than biofilm, and it has a large number of metabolites also at low concentrations, hence the lower R^2^. When the analysis is carried out with only those signals that have an S/*N* > 30, *R* ^2^ improves to 0.952 (Figure 6D). Acquisition of a data set with 64 scans and five planes took about 43 h, whereas acquiring only two planes requires only 17 h or even less when fewer scans are used by focusing on the metabolites with higher concentration. It should be noted that the first plane can be used as input for COLMARm for metabolite identification, as shown in Figure 6, thereby foregoing the measurement of a separate 2D HSQC experiment.

**Figure 6:**
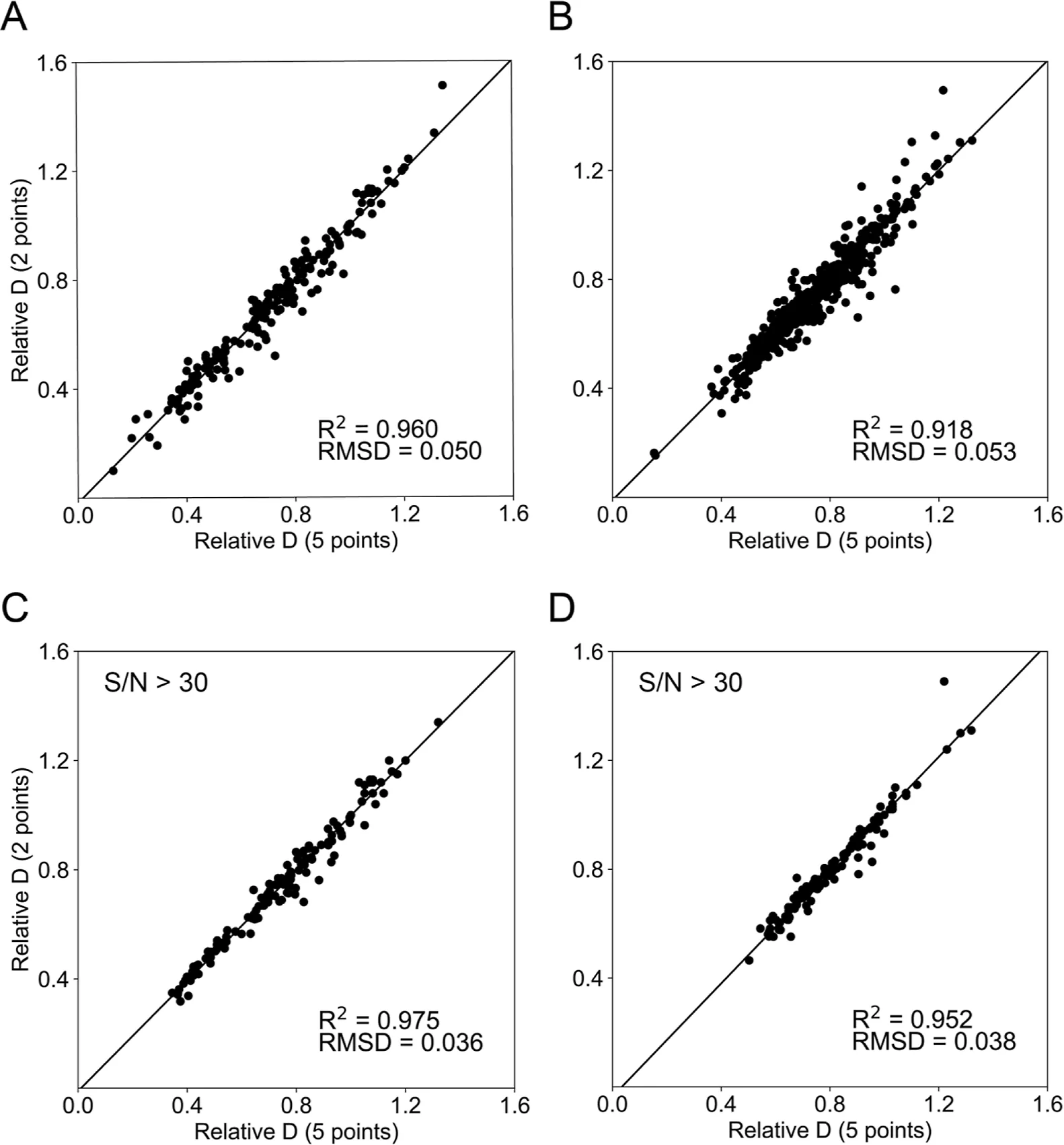
Correlation between the relative diffusion coefficients derived from the pure shift pseudo-3D HSQC-DOSY spectra of (A) biofilm and (B) mouse urine samples. Each data point represents a single ^1^H–^13^C HSQC cross-peak. The plots show a comparison between the diffusion values obtained by fitting all five gradient steps (*x*-axis) and those obtained from only two, namely the first and last gradient points (*y*-axis). Panels (C,D) display the correlations for the biofilm and mouse urine samples, respectively, considering only the cross-peaks with a signal-to-noise (S/N) ratio greater than 30. The *R* ^2^ coefficient of determination and the root-mean-square deviation (RMSD) are provided in each panel to quantify the agreement between the two methods.

The new real-time pure shift pseudo-3D HSQC-DOSY pulse sequence offers a significant increase in resolution and sensitivity, making the experiment suitable for applications to complex mixtures with a large number of molecular components and a broad range of concentrations typical for metabolomics samples. One of the motivations for this work was our observation that the common pseudo-2D DOSY experiment cannot adequately unravel the multiexponential decay behavior present in many overlapping peaks in our metabolomics samples for a meaningful interpretation. In addition to peak overlaps, baseline distortions and phase errors can adversely affect the fitting of diffusion coefficients in significant ways. Figure S9 shows the pseudo-2D DOSY spectra of biofilm and mouse urine, demonstrating that pseudo-2D DOSY is limited to a much lower number of peaks that can be reliably analyzed compared to the pure shift pseudo-3D DOSY experiment.

## Conclusion

The new real-time pure shift HSQC-DOSY experiment introduced here addresses challenges caused by low resolution and low sensitivity of existing DOSY pulse sequences in metabolomics applications. A comparison with the traditional HSQC-DOSY shows that the pure shift sequence yields significantly improved diffusion profiles and more precise diffusion coefficients, also for low concentration compounds. We demonstrated how this is directly beneficial for the correct identification of metabolites when their cross-peaks cannot be unambiguously assigned to a metabolite in the database. With the optional addition of nonuniform sampling and the ability to speed up the acquisition by acquiring a minimal number of planes for diffusion-coefficient determination, the new pseudo-3D DOSY experiment along with the COLMARvista DOSY-analysis module should become well-suitable for routine NMR metabolomics studies. The real-time pure shift pseudo-3D HSQC-DOSY pulse sequence is publicly available at https://github.com/RCabreraAllpas/Pulse-sequences.

## Supplementary Material



## Notes

Detailed DOSY parameters used for each experiment; improved diffusion fitting accuracy using pure shift pseudo-3D HSQC-DOSY; Voigt model fitting accuracy in the pure shift pseudo-3D HSQC-DOSY; comparison of diffusion coefficient values obtained by NUS and US pure shift pseudo-3D HSQC-DOSY experiments; comparison of representative peak fitting and diffusion coefficient analysis between NUS and US data sets; multiple COLMARvista DOSY fitting results for biofilm and mouse urine; examples where the standard HSQC-DOSY gives poor fits due to low S/N for certain cross-peaks; certain artifacts that can occur in the pure shift pseudo-3D HSQC-DOSY spectra; comparison between the relative diffusion coefficients when obtained by a 5-plane fitting vs a 2-plane fitting using a higher signal-to-noise threshold for mouse urine; comparison with standard pseudo-2D DOSY (PDF)
