# Supplementary material for: Fast Diffusion-Based Metabolomics Profiling by 3D Pure Shift HSQC-DOSY NMR with COLMARvista
Source: Anal Chem. 2026 Aug 11;98(33):24047–56. doi: 10.1021/acs.analchem.6c01028 (PMC13523553; doi:10.1021/acs.analchem.6c01028)
Supplement: Supplementary file 1 [file ac6c01028_si_001.pdf]

Supporting Information

Fast Diffusion-Based Metabolomics Profiling by 3D Pure Shift HSQC-DOSY NMR with COLMARvista

Cabrera Allpas, R.[a,b,+]; Lee, K.[a,+]; Li, D.-W.[a,*]; Choo, M.[a]; Bruschweiler-Li, L.[a]; Brüschweiler, R.[a,c,*]

[a] Department of Chemistry and Biochemistry, The Ohio State University, Columbus, Ohio 43210, United States.

[b] Department of Chemistry, University of North Carolina at Chapel Hill, Chapel Hill, North Carolina 27599, United States.

[c] Department of Biological Chemistry and Pharmacology, The Ohio State University, Columbus, Ohio 43210, United States.

[+] These authors contributed equally.

*Corresponding authors:

Rafael Brüschweiler E-mail: bruschweiler.1@osu.edu

Dawei Li E-mail: lidawei@gmail.com

Content of Supporting Information

- Detailed DOSY parameters used for each experiment.
- Improved diffusion fitting accuracy using pure shift pseudo-3D HSQC-DOSY.
- Voigt model fitting accuracy of pure shift pseudo-3D HSQC-DOSY.
- Comparison of diffusion coefficients obtained from NUS and US pure shift pseudo-3D HSQC-DOSY experiments.
- Comparison of representative peak fitting and diffusion coefficient analysis between NUS and US datasets.
- COLMARvista visualization of pseudo-3D DOSY fitting results for the biofilm.
- COLMARvista visualization of pseudo-3D DOSY fitting results from mouse urine.
- Cross-peaks comparison in pure shift vs. oneshot pseudo-3D HSQC-DOSY.
- Artifacts observed in pure shift pseudo-3D HSQC-DOSY spectra.
- Standard pseudo-2D DOSY plot of biofilm and mouse urine samples obtained with Dynamic Center 2.8.4.

Table S1. DOSY parameters used for the pulse programs used in this work.

Pulse program: pseudo-3D HSQC-DOSY (one-shot and pure shift)			
Sample/Parameter	δ (ms)	Δ (ms)	Gradient ramp (%)
Sucrose-glucose	1.70	79.99	5−80
Biofilm	1.60	79.99	5−80
Mouse urine	1.60	79.99	5−80
Pulse program: pseudo-2D DOSY			
Biofilm	1.70	79.99	5−95
Mouse urine	1.66	79.99	5−95

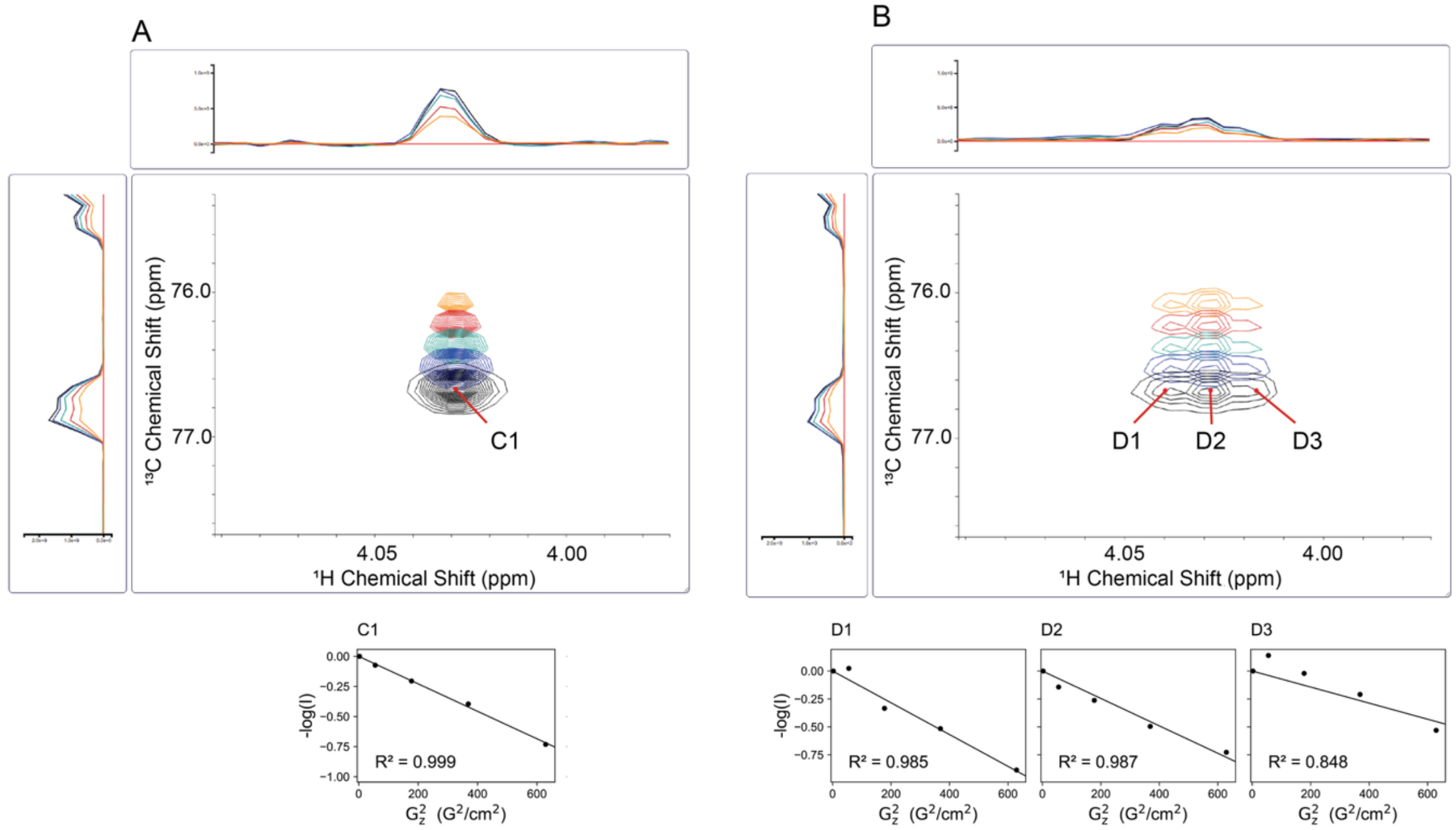

Figure S1. Comparison of (A) pure shift pseudo-3D HSQC-DOSY and (B) oneshot pseudo-3D HSQC-DOSY spectra focusing on the H-4'/C-4' cross-peak of the fructose unit in sucrose. These panels illustrate the utility of COLMARvista's multi-display feature, which facilitates the simultaneous analysis of the same spectral region across different experimental measurements. The signals shown in (A) and (B) correspond to peaks (C1) and (C2) in **Figure 2**, respectively. The bottom panels display signal decay and linear fits derived from 5 gradient planes. As shown in (A), the collapse of the triplet into a singlet using the pure shift method results in a better linear fit for the diffusion coefficient, compared to the individual triplet components (D1–D3) observed in the standard oneshot experiment in (B).

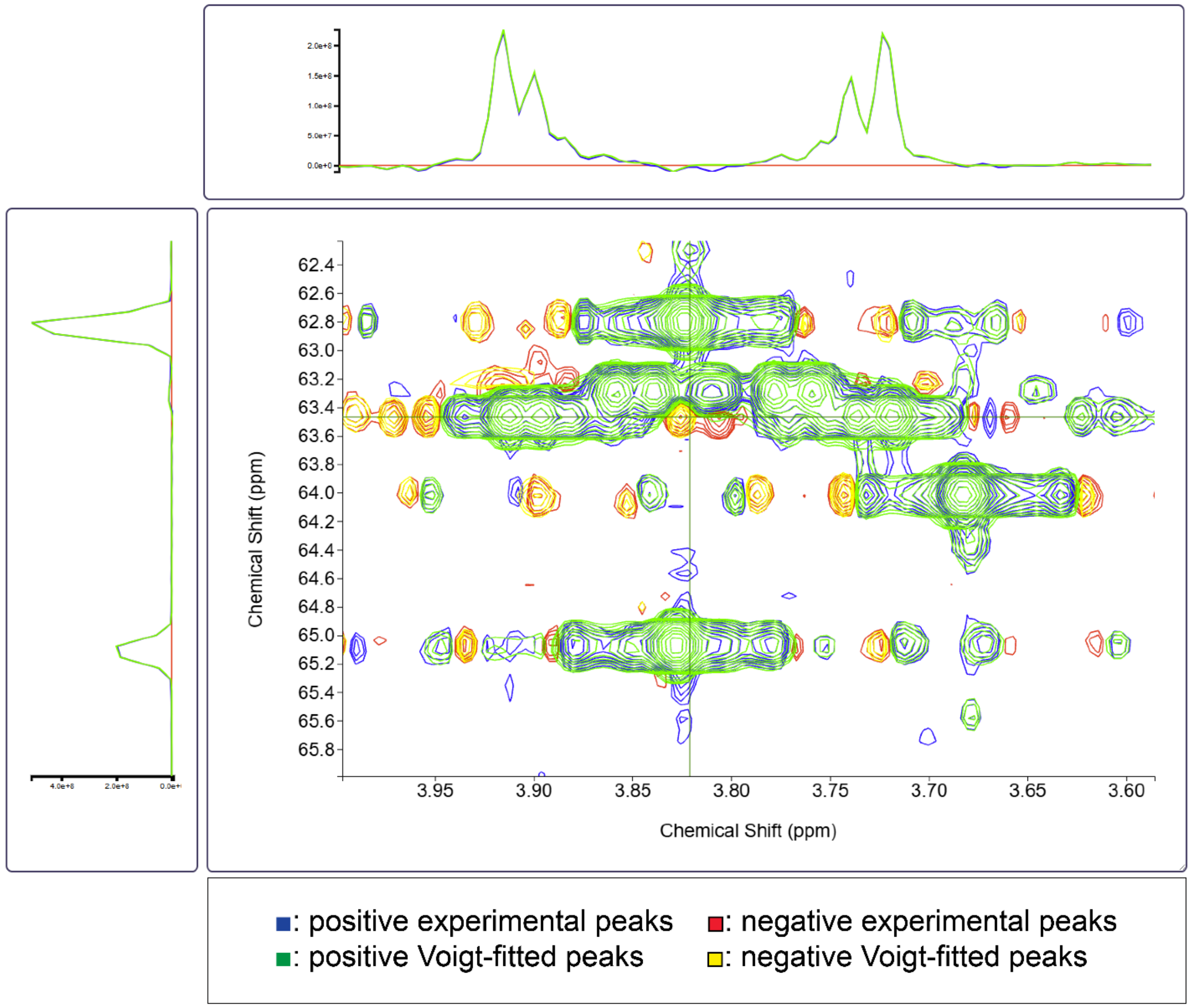

Figure S2. Experimental and Voigt-fitted pure shift pseudo-3D DOSY spectra of sucrose-glucose model mixture (2.5% w/v). Blue and red denote positive and negative experimental contours, respectively. Green and yellow denote positive and negative contours of the fitted Voigt peaks. The top and left panels show 1D cross-sections along the direct (^{1}H) and indirect (^{13}C) dimensions, respectively. The close overlap between the experimental and fitted contours indicates that the cross-peaks are well described by the Voigt model fitting. No obvious systematic lineshape distortions are observed that would compromise peak deconvolution and quantitative analysis.

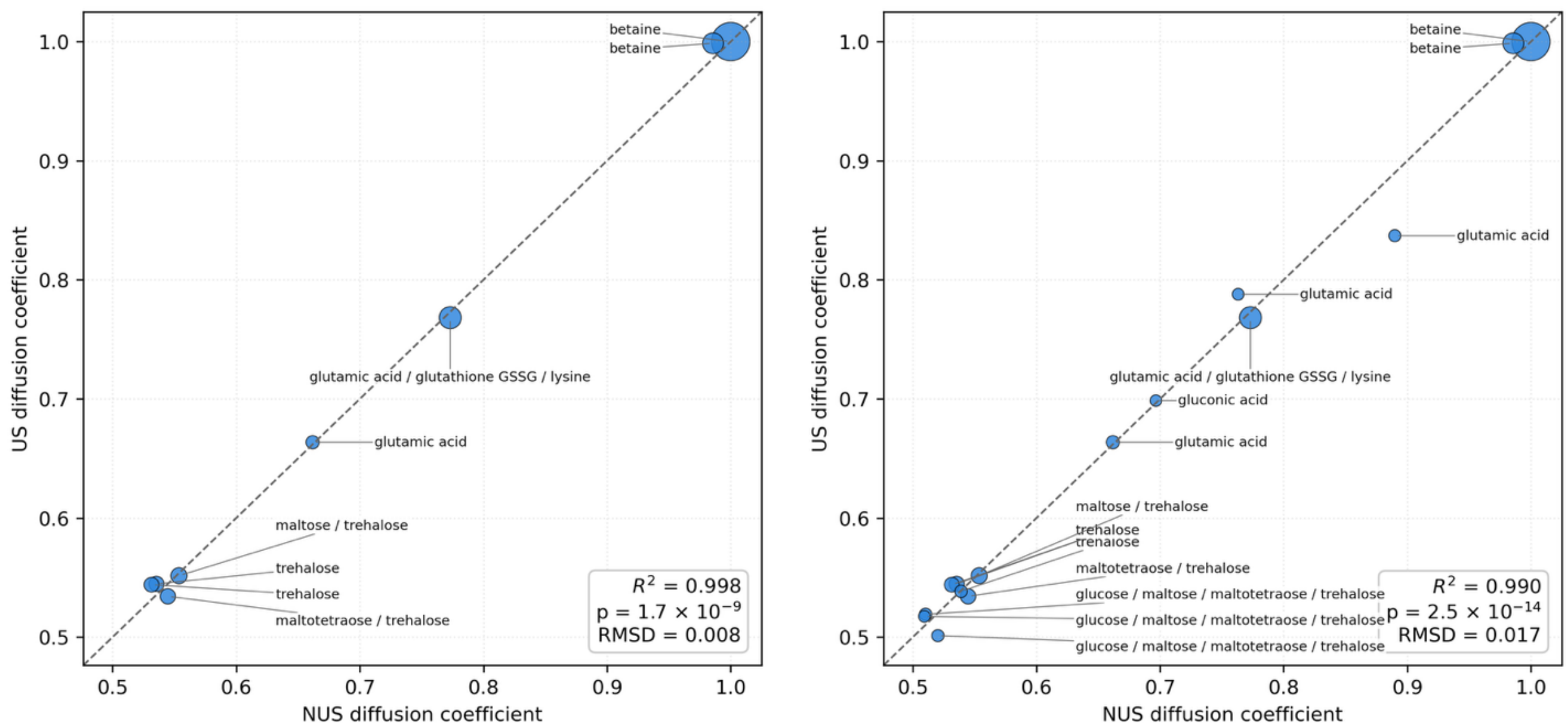

Figure S3. Comparison of diffusion coefficient values for matched peaks from the non-uniformly sampled (NUS) and uniformly sampled (US) pure-shift pseudo-3D HSQC-DOSY experiments of the biofilm sample. The US peak list was matched to the NUS peak list using both ^{1}H and ^{13}C chemical shifts. The left panel shows peaks with signal-to-noise ratios (S/N) greater than 120 in both datasets, and the right panel shows peaks with S/N greater than 80 in both datasets. Each bubble represents a matched peak pair, with the bubble size proportional to the average peak volume of the NUS and US pair. Assigned metabolites are indicated next to the bubbles, with multiple names denoting overlapping assignments.

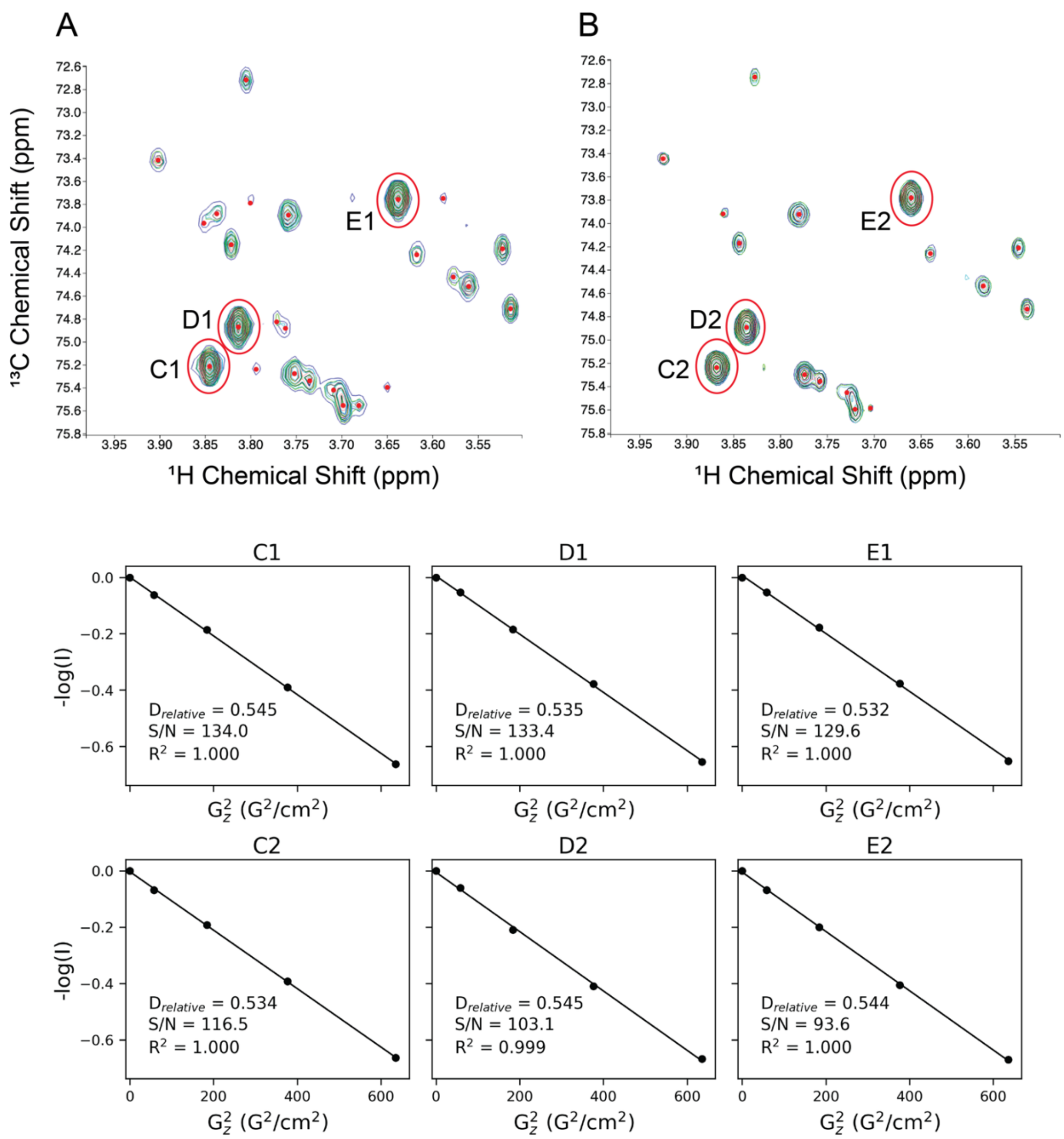

Figure S4. Trehalose-derived peaks used for fitting along with diffusion coefficient analysis in the pure shift pseudo-3D HSQC-DOSY spectra of the biofilm sample. The two top panels show the selected trehalose peaks in the NUS (A) and US (B) datasets and the lower panels show the diffusion decays along with fitting results (with R^2 values) labeled (C1), (D1), and (E1) for NUS and (C2), (D2), and (E2) for US. For each peak, the relative diffusion coefficient was calculated by normalizing its diffusion coefficient against that of betaine. The S/N was determined for each peak from the plane with the lowest gradient strength.

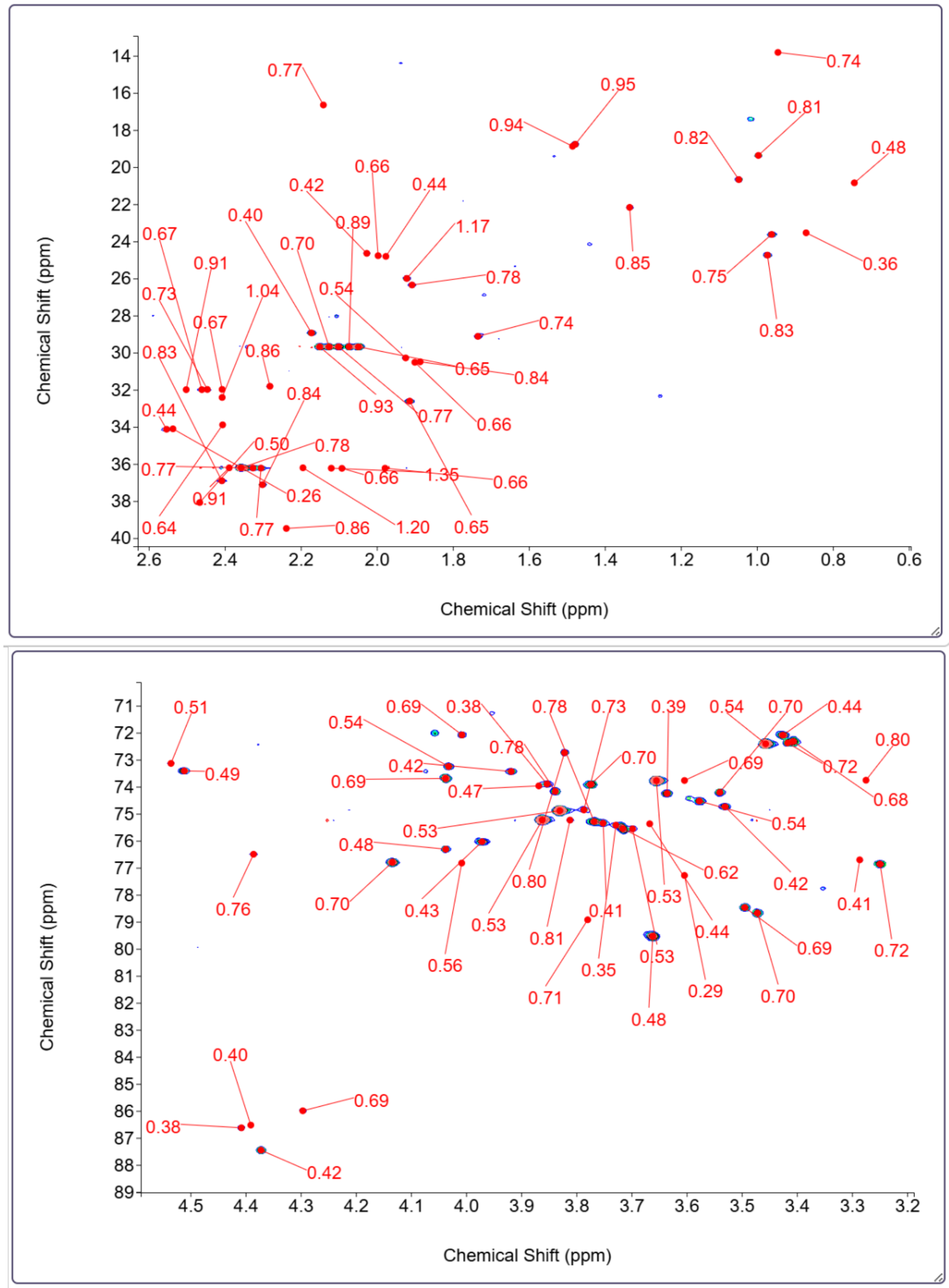

Figure S5. COLMARvista screenshots displaying the pure shift pseudo-3D HSQC-DOSY spectrum of the biofilm sample. All five acquired 2D planes are visualized as a color-coded stack plot, with the fitted relative diffusion coefficient annotated in red to each cross-peak. The relative diffusion coefficients were normalized to the diffusion coefficient of betaine using the equation: $D_{\text{relative}} = D_{\text{measured}} / D_{\text{Betaine}}$.

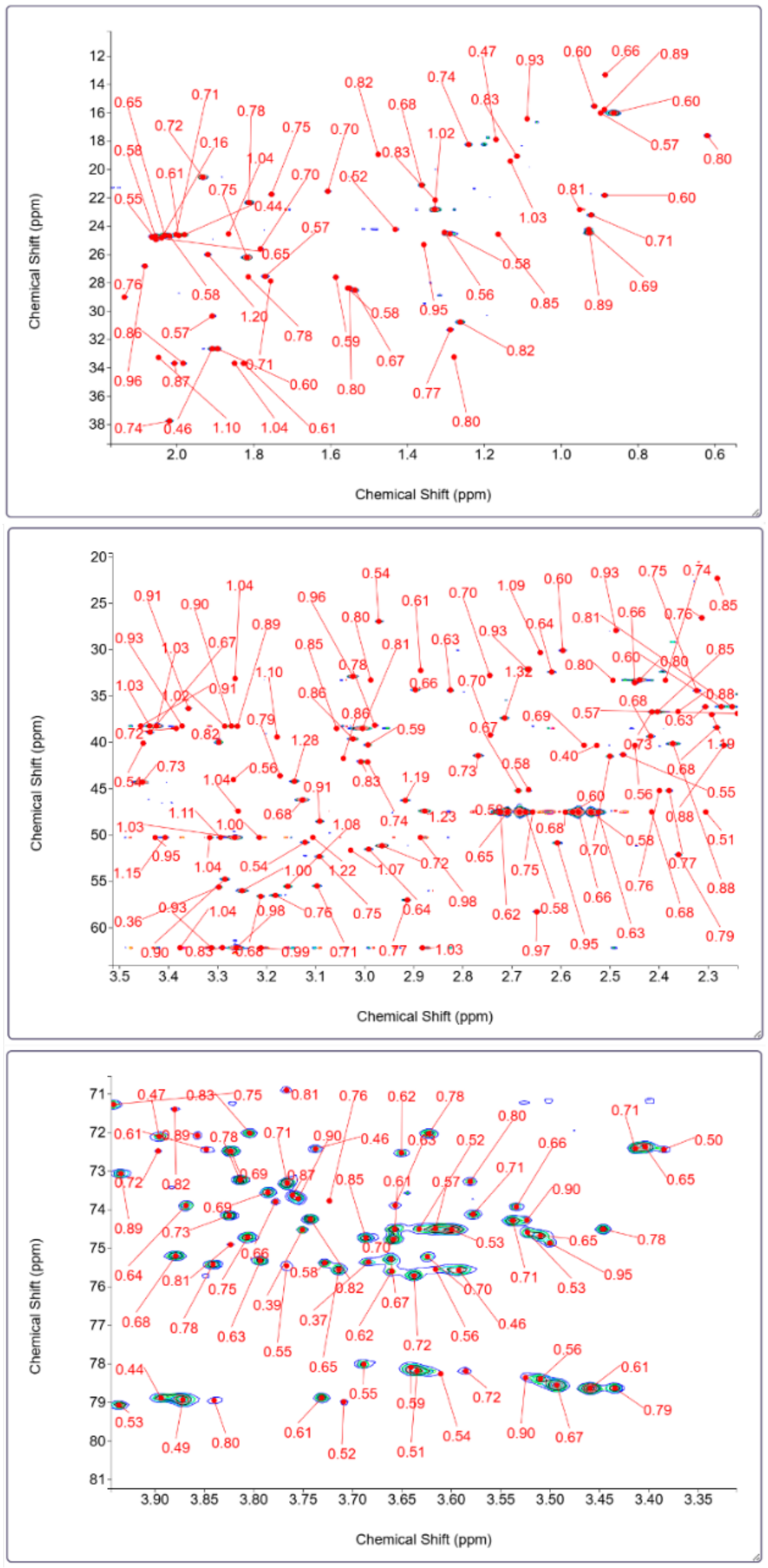

Figure S6. Screenshots from COLMARvista displaying three spectral regions of the pure shift pseudo-3D HSQC-DOSY spectrum obtained from a mouse urine sample. Fitting was performed directly within the web server using data from all five gradient planes. The resulting relative diffusion coefficients, using betaine as reference, are annotated in red next to each corresponding cross peak.

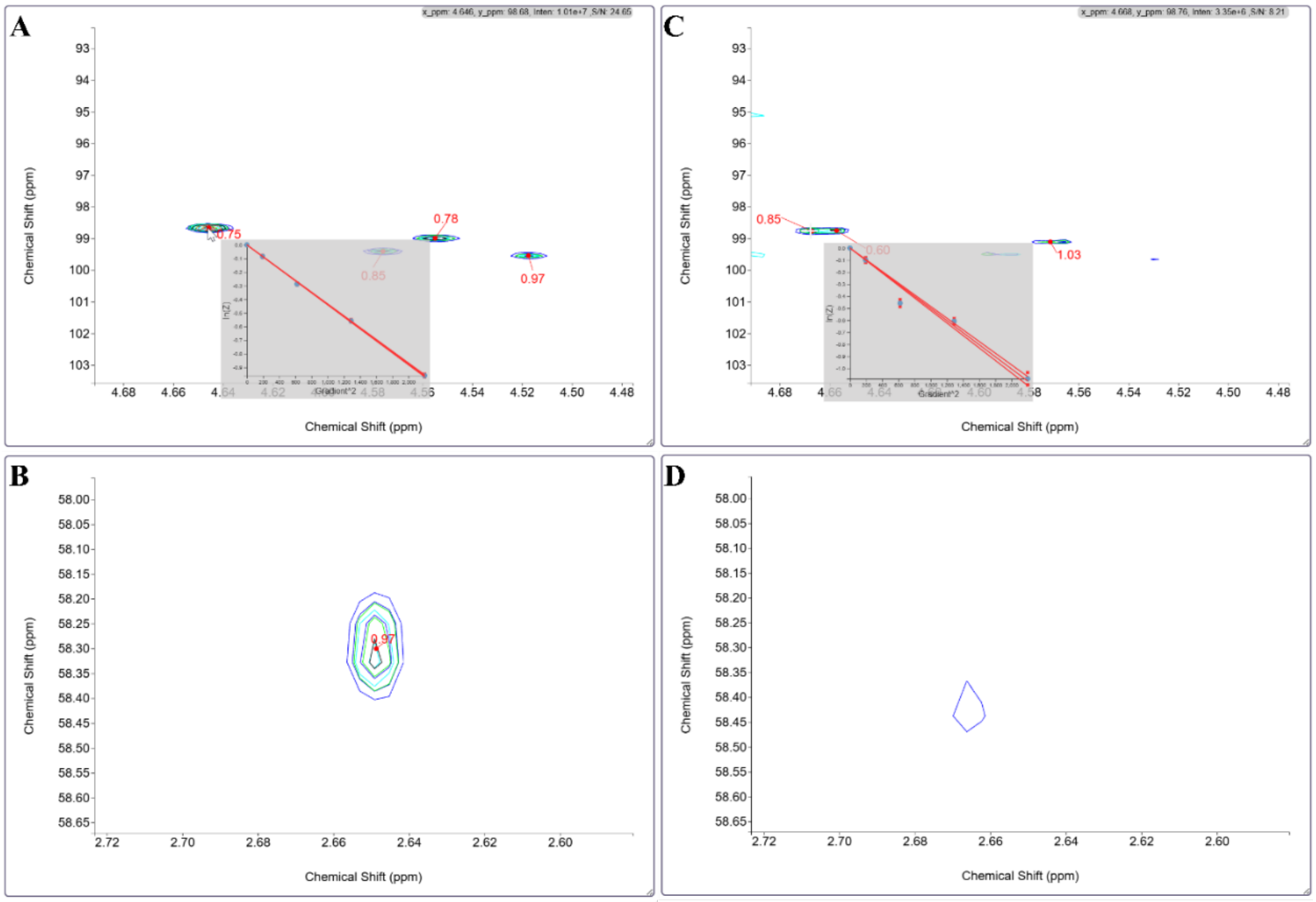

Figure S7. Comparison between (A, B) pure shift pseudo-3D HSQC-DOSY and (C, D) oneshot pseudo-3D HSQC-DOSY spectra obtained from a mouse urine sample, displayed by COLMARvista. Insets in (A) and (C) show diffusion decay curves and fitting for a cross-peak of β-glucose (at the position of the cursor). The fit obtained from the pure shift experiment in (A) exhibits better linearity compared to the oneshot experiment in (C), where several sugar-related cross-peaks show weak intensity, hindering accurate analysis. Panels (B) and (D) show a comparison of the hypotaurine cross-peak. In the oneshot spectrum (D), the signal is detectable only in the first gradient plane, preventing reliable determination of a diffusion coefficient. In contrast, the pure shift spectrum (B) retains sufficient signal intensity across all 5 planes, enabling quantitative diffusion analysis.

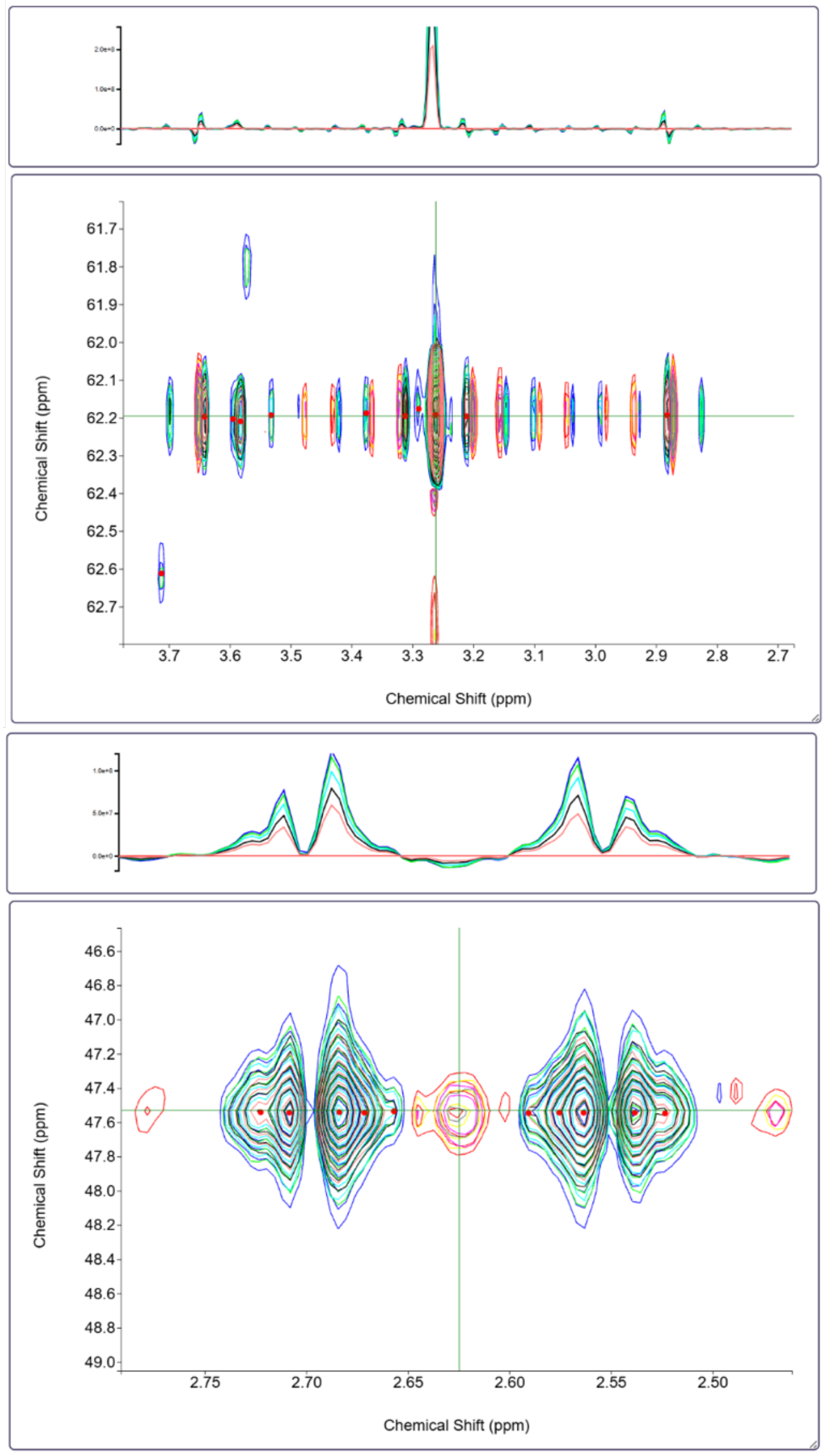

Figure S8. Representative artifacts observed in the pure shift pseudo-3D HSQC-DOSY experiments. Top Panel: Artifacts arising from chunking sidebands associated with strong signals. The top 1D plot displays a cross-section extracted from the 2D spectrum at the specific chemical shift indicated by the green crosshair. Bottom Panel: Artifacts resulting from the incomplete decoupling of diastereotopic methylene (CH_2) protons. Similarly, the 1D plot above shows the cross-section through the 2D spectrum at the position indicated by the green crosshair.

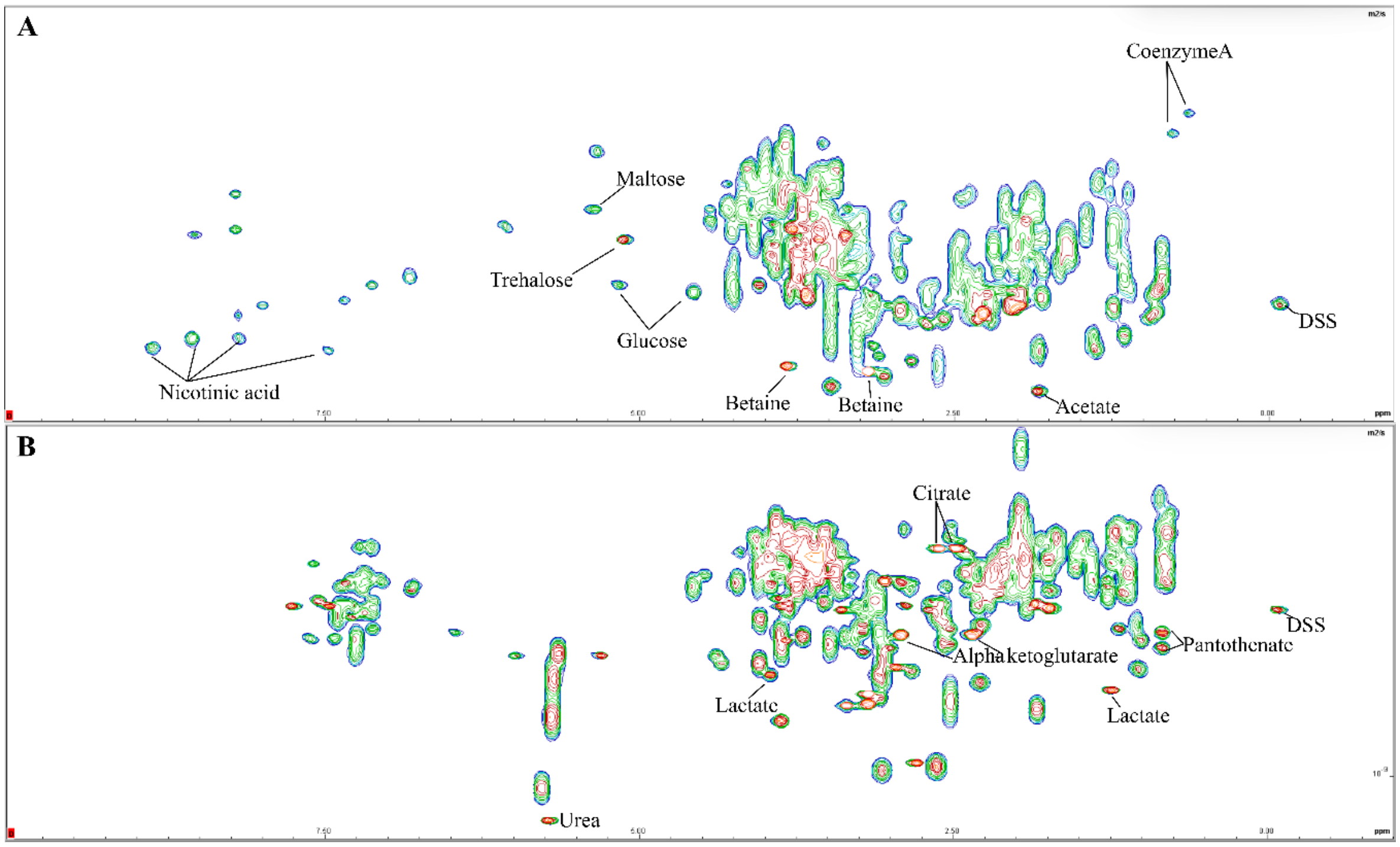

Figure S9. Pseudo-2D DOSY spectra of (A) biofilm and (B) mouse urine sample (^{1}H chemical shift along x-axis and diffusion coefficient D along y-axis). Relatively few metabolites with isolated signals can be uniquely identified and analyzed, whereas most metabolites fall in congested spectral regions preventing the accurate quantitative analysis due to peak overlap and baseline distortions. The plots were generated using Dynamics Center 2.8.4 (Bruker Biospin, Rheinstetten, Germany).
